# Evaluation of and the prognostic factors for cats with big kidney‐little kidney syndrome

**DOI:** 10.1111/jvim.16279

**Published:** 2021-10-15

**Authors:** Yen‐Tse Wu, Wan‐Chu Hung, Po‐Yao Huang, Han‐Ju Tsai, Ching‐Ho Wu, Ya‐Jane Lee

**Affiliations:** ^1^ Institute of Veterinary Clinical Science, School of Veterinary Medicine, College of Bio‐Resources and Agriculture National Taiwan University Taipei Taiwan; ^2^ National Taiwan University Veterinary Hospital, College of Bio‐Resources and Agriculture National Taiwan University Taipei Taiwan; ^3^ Department of Emergency and Critical Care Auburn University Veterinary Teaching Hospital Auburn Alabama USA

**Keywords:** AKI, CKD, feline, ureteral obstruction

## Abstract

**Background:**

The term big kidney‐little kidney syndrome in cats has been used for many years, but the definitions are not consistent and relevant research is limited.

**Objective:**

To determine the factors that differ between normal and BKLK cats, as well as to develop models for predicting the 30‐day survival of cats with ureteral obstruction (UO).

**Animals:**

Sixteen healthy cats and 64 cats with BKLK.

**Methods:**

Retrospective study. To define BKLK by reference to data from clinically healthy cats. The demographic and clinicopathological data among groups were statistically analyzed.

**Results:**

Big kidney‐little kidney syndrome cats had higher blood urea nitrogen (BUN) (median [interquartile range] 69 [28‐162] vs 21 [19–24] mg/dL, *P* < .001), creatinine (5.6 [1.9‐13.3] vs 1.3 [1.05‐1.40] mg/dL, *P* < .001), and white blood cells (10 800 [7700‐17 500] vs 6500 [4875‐9350] /μL, *P* < .001) and lower hematocrit (32.8 [27.1‐38.4] vs 39.1 [38.1‐40.4]%, *P* < .001), urine specific gravity (1.011 [1.009‐1.016] vs 1.049 [1.044‐1.057], *P* < .001) and pH (5.88 [5.49‐6.44] vs 6.68 [6.00‐7.18], *P* = .001) compared to the control cats. A lower body temperature (BT; 38.1 [37.9‐38.2] vs 38.7 [38.3‐39.2]°C, *P* = .009), higher BUN (189 [150‐252] vs 91 [36‐170] mg/dL, *P* = .04), and creatinine (15.4 [13.3‐17.4] vs 9.0 [3.1‐14.2] mg/dL, *P* = .03) were found among the UO cats that were not 30‐day survivors. A combination of BUN, phosphorus, and BT can predict 30‐day survival among UO cats with an area under receiver operating characteristic curve of 0.863. (*P* = .01).

**Conclusion:**

An increase in the length difference between kidneys can indicate UO, but cannot predict outcome for BKLK cats.

**Abbreviations**
AUROC: area under receiver operating characteristic curve
BKLKS: big kidney‐little kidney syndrome
BT: body temperature
BUN: blood urea nitrogen
CKD: chronic kidney disease
IQR: interquartile range
L2: second lumbar vertebra
LK: left kidney
NLR: neutrophil‐to‐lymphocyte ratio
Non‐UO: nonureteral obstruction
OR: odds ratio
RK: right kidney
ROC: receiver operating characteristic
SI: surgical intervention
SUB: subcutaneous ureteral bypass system
UO: ureteral obstruction
UpH: urine pH
USG: urine specific gravity
WBCs: white blood cells

## 1 INTRODUCTION

The term Big Kidney‐Little Kidney Syndrome (BKLKS) was first suggested in 2011 in Nephrology and Urology of Small Animals.^1^ The term describes an extreme asymmetry in size of the kidneys in a single cat. This has been often recognized to be the result of a unilateral ureteral obstruction (UO),^1^ and be the cause of subsequent changes in renal structures including atrophy, fibrosis, or both, of the affected kidney as well as a compensatory hypertrophy of the contralateral kidney. These effects are the most frequent causes of the clinical presentation of a large kidney and a little kidney in a cat.^1^

Cats are predisposed to UO as it could be due to their small ureteral lumen,^2^ and a high incidence of UO has been recorded in cats over recent decades.^3^ In cats, most UOs are secondary to calcium oxalate uroliths,^4^ but can also be caused by tumors, strictures, iatrogenic ligation, surgical trauma, mucus, mucopurulent plugs, blood clots, and dried solidified blood calculus.^4^, ^5^, ^6^, ^7^ Ureteral obstruction in most cases is initially unilateral; however, animals often present for medical care with bilateral UOs, or with a unilateral obstruction and concurrent contralateral kidney dysfunction due to any cause of reduced renal function including previous UO.^4^

When UO occurs, it increases hydraulic pressure within the ureter and renal pelvis, and if high enough, this is transmitted to the tubules and Bowman's space, reducing glomerular filtration rate. If the contralateral renal function is preserved, the cat does not usually become azotemic,^5^ and the episodes of the disease might easily be overlooked by the cats' owners. The UO can be dynamic and even resolve spontaneously.^8^ When the obstruction is unresolved, however, whether it is either complete or partial, this will affect the functioning of the kidneys, as well as their structure and this then leads to the BKLKS.

BKLKS is sometimes an incidental finding and such cats are typically stable and often in clinical practice do not show signs of azotemia.^5^ However, cats might be presented with uremia, either where both kidneys appear to be chronically compromised, or where it appears that 1 kidney has undergone compensatory hypertrophy and then becomes obstructed.^5^ The latter can result in severe azotemia and be accompanied by life‐threatening hyperkalemia.^5^ As there is no consistent definition of BKLKS in veterinary medicine at present, the primary objectives of this study are to describe the characteristics of cats with BKLKS. Next, we aimed to define the correlation between the extent of BKLKS and various relevant clinical and clinicopathological values, as well as various radiographic and ultrasonographic features. Finally, we wanted to determine any potential prognostic factors for cats with BKLKS.

## 2 MATERIALS AND METHODS

### 2.1 Criteria for diagnosing BKLKS

Sixteen clinically healthy cats underwent a complete physical examination and were found to have a normal abdominal radiographic examination, a normal blood count, and normal biochemistry values, as well as normal urinalysis results; these control cats were enrolled and used for the determination of the criteria of diagnosing BKLKS. All the cats had creatinine concentration lower than 1.6 mg/dL, and all had plasma total thyroxine concentrations within normal limits. The length of both kidneys on an abdominal ventrodorsal radiograph was measured and the kidney length difference of each cat was recorded. The length of the right kidneys (RKs) ranged from 3.31 to 5.90 cm with a median of 4.37 cm. The length of the left kidney (LK) ranged from 3.48 to 5.87 cm with a median of 4.54 cm. The length differences for the control group ranged from 0.01 to 0.69 cm with a median of 0.23 cm. Based on these findings, we defined the upper limit of this range as the cutoff value for determining BKLKS in this study. The largest value for length difference among the control cats was 0.69 cm and, after taking observational error into account, we settled on 0.70 cm as the cutoff value. The criterion for BKLKS in this study was thus defined as a cat having a length difference between their 2 kidneys on abdominal ventrodorsal radiograph that exceeded 0.70 cm.

### 2.2 Cases selection and grouping

For this retrospective study, radiograph records at the National Taiwan University Veterinary Hospital were reviewed. The inclusionary criterion for BKLKS is a length difference between the kidneys exceeding 0.7 cm when measured on abdominal ventrodorsal radiograph. Cases that fulfilled the inclusion criterion were identified. Cases were excluded if a renal mass, polycystic kidney disease, a perinephric pseudocyst, feline infectious peritonitis, or any neoplastic disease involving other body organs was highly suspected.

**FIGURE 1:**
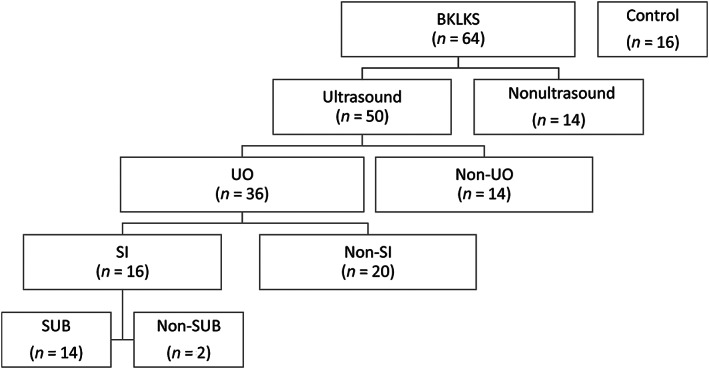
Summary of the diagnostic categories and management of 64 cats with big kidney‐little kidney syndrome. BKLKS, patients with big kidney‐little kidney syndrome; SI, patient with surgical intervention; SUB, patient with subcutaneous ureteral bypass surgery; Ultrasound, patients who underwent ultrasonographic examination; UO, patients with ureteral obstruction

The selected cases were first separated into 2 groups based on whether an abdominal ultrasound examination had been performed. Cats with an abdominal ultrasound examination were later divided into 2 groups based on whether or not an episode of UO was present at presentation to the hospital. In this study, cats were grouped into the UO group mainly based on the presence of hydronephrosis (the objective length of pelvis in transverse section exceeding 0.35 cm) or hydroureter (the diameter of the proximal ureter exceeding 0.2 cm on ultrasound examination), with or without hyperechogenic materials detected in the ipsilateral ureteral outflow tract, with concomitant history. Relevant history included various acute clinical signs such as vomiting, anorexia, lethargy, dehydration, pain on abdominal palpation, weight loss, oliguria/anuria or polydipsia with polyuria, and the cat's clinicopathological results, such as an increased serum creatinine value. Cats diagnosed with UO were further divided into 2 groups based on whether the UO was treated by surgery including subcutaneous ureteral bypass (SUB) or a ureteral stent (Figure 1).

### 2.3 Data collection

The data for this study included a range of clinicopathological data as well as medical images. These consisted of signalment, any previous history of nephrological diseases, the physical examination findings, the radiographic examinations, ultrasound examinations, complete blood counts with a blood film evaluation and a manual (white blood cells [WBCs]) differential count, plasma biochemical profiles, urinalysis (with the urine pH [UpH] measured by a pH meter), and outcome.

The measurement of the difference in the size of each cat's kidneys and an examination of their structures were all obtained from an abdominal ventrodorsal view radiography. These included length of the second lumbar vertebral body (L2), and the length of individual kidneys. Kidney length differences, kidney‐to‐L2 ratio, and the differences in ratio were calculated and then recorded. The ultrasound results for the lengths of the individual kidneys were recorded in the sagittal or horizontal plane, which is where the longest kidney length could be observed. Additionally, the degree of pelvic or ureteral dilatation, if present, was also recorded.

The outcome for each cat was tracked and recorded for up to 1825 days after initial presentation and the cats with UO were evaluated as either survivors or nonsurvivors based on their survival for 30 days.

### 2.4 Statistical analysis

Statistical analysis was performed using a statistical software package (SPSS 25.0 for Mac). The Shapiro‐Wilk test was used to determine the normal distribution condition for the continuous variables. The Mann‐Whitney test and Kruskal‐Wallis test (post hoc with the Dunn test) were used for comparisons between or among continuous variables, while Pearson chi‐square was used for categorical variables. Linear regression analysis was used to evaluate the relationship between 2 variables. Spearman correlation test was used to evaluate 2 nonparametric variables. Logistic regression analysis was used for calculating odds ratios (ORs) for univariate and multivariates.

Receiver operating characteristic (ROC) curve analyses were used to test the ability of variables to predict if a kidney was obstructed and/or if the obstructed cats survived as well as to establish cutoff values for such predictions.

Kaplan‐Meier curves were generated to assess survival; this was done using the cutoff values established from the previous ROC curve results, and then tested them using log‐rank tests. Differences of *P* < .05 were considered significant for the selected variables with a 95% confidence interval.

## 3 RESULTS

In total, 64 cats that met the inclusion criteria were enrolled (Figure 1); these consisted of 38 (59%) domestic shorthairs, 6 (9%) American Shorthair cats, 5 (7%) Chinchillas, 4 (6%) Scottish folds, 3 (4%) Persians, 2 (3%) Abyssinians, 2 (3%) British Shorthairs, 1 (1%) Ragdoll, and 3 (4%) cats with unrecorded breeds.

Based on the ventrodorsal abdominal radiographs, in addition to having a greater difference in length (median, interquartile range [IQR]; 1.38 [0.97‐2.00] vs 0.23 [0.05‐0.28] cm, *P* < .001), BKLKS cats also had 1 kidney with increased absolute length (5.11 [2.51‐5.52] vs 4.55 [4.11‐4.79] cm, *P* = .002), and increased kidney‐to‐L2 ratio (2.59 [2.40‐2.84] vs 2.37 [2.21‐2.63], *P* = .03) compared to the control cats. However, the L2, LK, and RK length had no significant differences between the 2 groups (Table 1). There were 33 out of 64 cats (51%) in the BKLKS group whose LK was the larger 1, while 31 out of 64 (48%) cats had their RK being the larger 1. Radiographically, there were 15 (23%) cats with nephrolith alone, 12 (18%) cats with nephrolith and ureterolith, 2 (3%) with nephrolith and cystolith, and 1 (1%) cat with only cystolith.

**TABLE 1:** Selected variables among the control cats, all BKLKS cats, the nonobstructed BKLKS cats, and the obstructed BKLKS cats

Parameters	Control (n = 16)		All BKLKS (n = 64)		*P* value^*^	Non‐UO BKLKS (n = 14)		UO BKLKS (n = 36)		*P* value^†^
	Median (IQR)	n	Median (IQR)	n		Median (IQR)	n	Median (IQR)	n	
Age (y)	8.00 (7.00‐11.50)	16	9.00 (6.00‐11.00)	64	.7	9.50 (7.75‐12.00)	14	8.00 (6.00‐11.00)	36	.69
BW (kg)	4.65 (3.50‐6.07)	12	4.08 (3.34‐5.26)	63	.26	4.22 (3.62‐5.85)	13	3.91 (3.39‐5.32)	36	.47
Sex	(8M, 8F)	16	(36M, 28F)	64	.65	(8M, 6F)	14	(20M, 16F)	36	.92^§^
BT (°C)	—	—	38.4 (38.0‐38.9)	41	—	38.7 (37.4‐39.3)	5	38.5 (38.1‐38.9)	27	.82^‡^
Length of L2 (cm)	1.89 (1.74‐2.04)	16	1.96 (1.84‐2.08)	64	.12	1.96 (1.88‐2.18)	14	1.96 (1.84‐2.05)	36	.56
Length diff. (cm)	0.23 (0.05‐0.28)^a^	16	1.38 (0.97‐2.00)	64	<.001	1.15 (0.95‐1.37)^b^	14	1.72 (1.27‐2.33)^c^	36	<.001
Ratio diff.	0.12 (0.02‐0.16)^a^	16	0.75 (0.51‐1.01)	64	<.001	0.59 (0.49‐0.66)^b^	14	0.86 (0.62‐1.21)^c^	36	<.001
BK length (cm)	4.55 (4.11‐4.79)^a^	16	5.11 (2.51‐5.52)	64	.002	5.03 (4.62‐5.32)^a,b^	14	5.23 (4.84‐5.75)^b^	36	.001
BK‐to‐L2 ratio	2.37 (2.21‐2.63)^a^	16	2.59 (2.40‐2.84)	64	.03	2.48 (2.34‐2.65)^a,b^	14	2.66 (2.48‐2.89)^b^	36	.005
Hematocrit (%)	39.1 (38.1‐40.4)^a^	16	32.8 (27.1‐38.4)	63	<.001	33.8 (30.4‐41.0)^a,b^	14	30.4 (24.2‐35.6)^b^	35	<.001
Platelets (10^3^/μL)	223 (142‐299)	16	273 (224‐348)	63	.05	285 (165‐389)	14	281 (237‐348)	35	.09
WBCs (/μL)	6550 (4875‐9350)^a^	16	10 800 (7700‐17 500)	63	<.001	9950 (6550‐15 550)^a,b^	14	10 600 (7700‐17 500)^b^	35	.006
Neutrophils (/μL)	4419 (2421‐6451)^a^	16	9048 (5854‐15 694)	61	<.001	7553 (4456‐14 195)^b^	13	8824 (6007‐15 792)^b^	34	.001
Lymphocytes (/μL)	1710 (954‐2180)	16	990 (602‐1623)	61	.03	990 (593‐1331)	13	959 (471‐1751)	34	.06
Eosinophils (/μL)	534 (300‐890)^a^	16	180 (0‐538)	60	<.001	500 (0‐926)^a,b^	13	180 (0‐326)^b^	33	.001
NLR	2.67 (1.37‐5.43)^a^	16	7.91 (4.30‐20.18)	61	<.001	6.70 (2.85‐23.38)^b^	13	10.32 (4.29‐23.89)^b^	34	.001
BUN (mg/dL)	21.5 (19.0‐24.5)^a^	16	69 (28‐162)	64	<.001	21.0 (18.3‐53.8)^a^	14	101.0 (49.3‐198.0)^b^	36	<.001
Creatinine (mg/dL)	1.30 (1.05‐1.40)^a^	16	5.6 (1.9‐13.3)	64	<.001	1.85 (1.58‐4.18)^b^	14	10.35 (3.80‐16.45)^c^	36	<.001
Albumin (g/dL)	3.10 (2.90‐3.28)	16	3.2 (3.0‐3.5)	61	.27	3.10 (3.05‐3.45)	13	3.20 (2.80‐3.40)	34	.56
Total protein (g/dL)	7.40 (7.10‐7.98)	16	7.3 (6.9‐8.0)	56	.54	7.30 (6.80‐7.75)	13	7.25 (6.80‐8.03)	30	.7
Glucose (g/dL)	134.5 (110‐161)	16	138 (108‐150)	13	.85	137 (108‐146)	14	120 (108‐160)	28	.8
K+ (mmol/L)	—	—	3.91 (3.54‐4.65)	63	—	3.44 (3.05‐3.75)	14	4.40 (3.77‐5.20)	35	<.001^‡^
ALKP (U/L)	44.5 (38.5‐48.0)^a^	16	34 (25‐44)	57	.006	38.0 (30.5‐81.0)^a^	14	30.0 (24.0‐38.8)^b^	30	.001
USG	1.049 (1.044‐1.057)^a^	16	1.011 (1.009‐1.016)	34	<.001	1.010 (1.009‐1.030)^b^	7	1.010 (1.009‐1.012)^b^	22	<.001
Urine pH	6.68 (6.00‐7.18)^a^	16	5.88 (5.49‐6.44)	34	.001	6.15 (5.76‐6.40)^a,b^	7	5.82 (5.49‐6.48)^b^	22	.01
Survival time (d)	—	—	255 (56‐602)	64	—	204 (113‐773)	14	272 (56‐813)	36	1^‡^

*Note*: Superscript letters (a, b, c): Within a row, values with different superscripts differ significantly (*P* < .05).

Abbreviations: ALKP, alkaline phosphatase; BK, big kidney; BKLKS, big kidney‐little kidney syndrome; BT, body temperature; BUN, blood urea nitrogen; BW, body weight; F, female; IQR, interquartile range; K+, potassium; L2, second lumbar vertebra; M, male; NLR, neutrophil‐to‐lymphocyte ratio; Non‐UO, nonureteral obstruction; ratio diff., the difference between the kidneys to second lumbar vertebra ratios; RBC, red blood cells; UO, ureteral obstruction; USG, urine specific gravity; WBCs, white blood cells.

^*^ *P* value generated by Mann‐Whitney test, between control cats and all BKLKS cats.

^†^ *P* value generated by Kruskal‐Wallis test (post hoc with Dunn test), among control cats, nonobstructed BKLKS cats, and obstructed cats.

^‡^ *P* value generated by Mann‐Whitney test, between nonobstructed BKLKS cats, and obstructed cats.

^§^ *P* value generated by chi‐square test, between nonobstructed BKLKS cats, and obstructed cats.

When the BKLKS cats were compared to the control cats, there were no significant difference in age (*P* = .7) and sex (*P* = .65) distribution between the 2 groups, but BKLKS cats had significantly higher blood urea nitrogen (BUN) concentrations (*P* < .001), plasma creatinine concentrations (*P* < .001), WBCs (*P* < .001), neutrophil counts (*P* < .001), neutrophil‐to‐lymphocyte ratios (NLRs) (*P* < .001), and monocyte counts (*P* = .04). They also had lower alkaline phosphatase activities (*P* = .006), hematocrits (*P* < .001), eosinophil counts (*P* < .001), and lymphocyte counts (*P* = .03) (Table 1).

Urinalysis was performed on 34 (53%) of the BKLKS cats. The BKLKS group were found to have significantly lower urine specific gravity (USG; *P* < .001) as well as a lower UpH (*P* = .004) when compared to the control cats (Table 1).

Overall, 50 out of the 64 BKLK cats (73%) underwent an ultrasonographic examination. The median LK length was 3.67 cm (ranging from 1.74 to 6.77 cm, n = 48), and median RK length was 3.62 cm (ranging from 2.23 to 6.53 cm, n = 48). In total, 36 out of the 50 cats that underwent abdominal ultrasonography (72%) were diagnosed with UO by either hydronephrosis or hydroureter based on their ultrasonographic findings, with 29 out of 36 (80%) having only hydronephrosis noted, 2 out of 36 (5%) with only hydroureter, and 5 out of 36 (13%) having both. The median pelvic dilatation in the obstructed cats was 1.02 cm (range, 0.22‐4.30 cm, n = 34), and the ureteral dilatation in the obstructed cats was 0.44 cm (range, 0.32‐0.89 cm, n = 7).

The obstructed BKLKS cats, when compared with nonobstructed BKLKS cats, had significantly larger length differences (median 1.72 cm vs 1.15 cm, *P* = .009), and ratio differences between their kidneys, (median 0.86 vs 0.59, *P* = .008), higher plasma creatinine concentrations (median 10.35 mg/dL vs 1.85 mg/dL, *P* < .001), higher BUN concentrations (median 101.0 mg/dL vs 21.0 mg/dL, *P* < .001), and higher potassium concentrations (median 4.40 mmol/L vs 3.34 mmol/L, *P* < .001), as well as lower alanine transaminase activities (median 39 U/L vs 68 U/L, *P* = .03). The median survival time was not significantly different between the obstructed BKLK cats (272 days, IQR 56‐813 days) and the nonobstructed BKLK cats (204 days, IQR 113‐773 days, *P* = 1) (Table 1).

**FIGURE 2:**
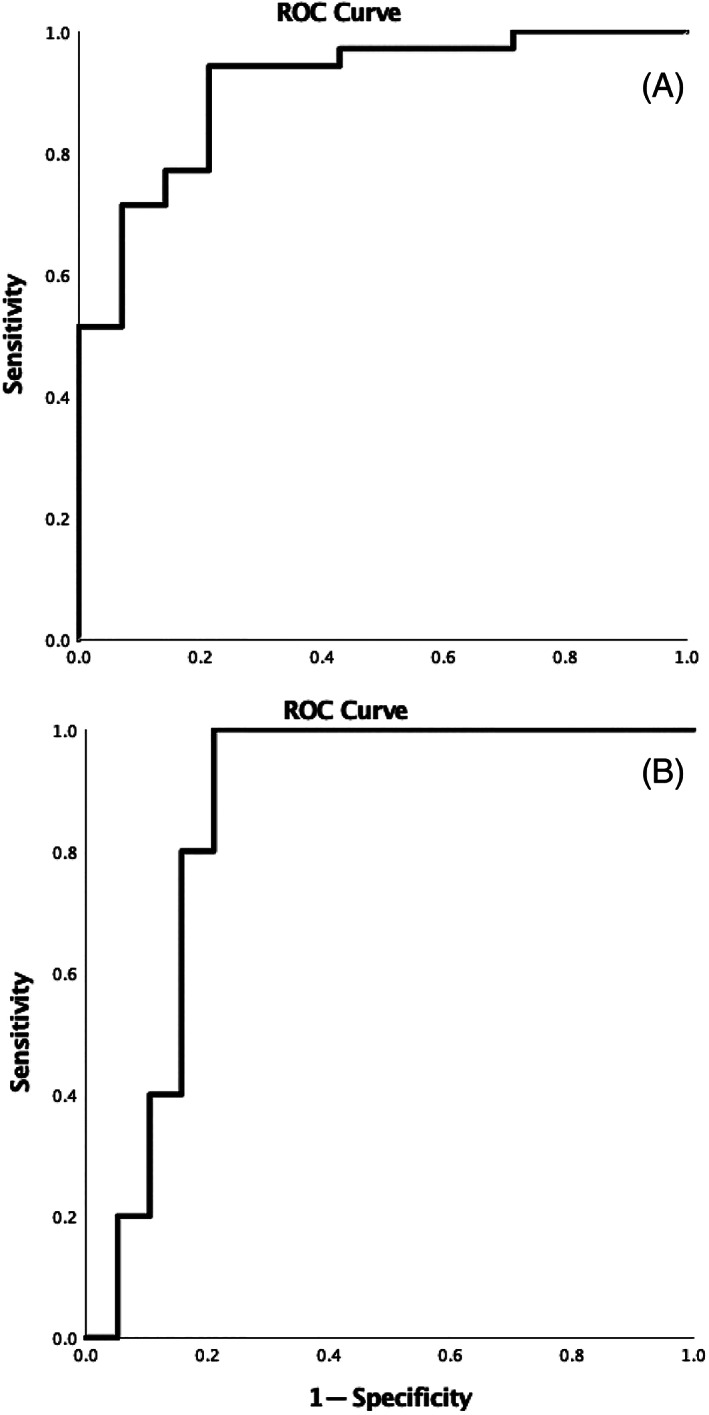
Receiver‐operating characteristic plots of diagnostic tests for ureteral obstruction using the formula [1.2 Creatinine (mg/dL) + 7.0 Potassium (mmol/L) + 18.0 Difference between the kidney‐to‐L2 ratios] (A). The formula which was designed as a diagnostic test for 30‐day survival in obstructed cats [1.0 BUN (mg/dL) + 0.8 Phosphorus (mg/dL) + 0.9 Body temperature (°C)] was presented with the ROC plot in (B). The area under the ROC (AUROC) for (A) is 0.908 (*P* < .001). Using a cutoff point of 41.3, for (A), the sensitivity was 94.3% and the specificity was 78.6%. The AUROC for (B) was 0.863 (*P* = .01). Using a cutoff point of 166.9, the sensitivity was 100% and the specificity was 78.9%. BUN, blood urea nitrogen; ROC, receiver operating characteristic

The univariate analysis using logistic regression showed that increased creatinine (OR = 1.296), increased BUN (OR = 1.019), increased potassium (OR = 5.729), and a higher ratio difference between the 2 kidneys (OR = 16.60) were significantly associated with the presence of UO. When kidney length difference alone was used as a variable to detect UO, it had an area under ROC (AUROC) of 0.740 (*P* = .009). When multivariate analysis was performed using logistic regression, multiple variable combinations in different models were tested (Table S1), and a formula with the optimal AUROC was derived from the multivariate analysis with creatinine and potassium concentrations, and the difference between the kidney‐to‐L2 ratios being the variables: [Log (odds of obstruction) = 1.188 Creatinine (mg/dL) + 7.286 Potassium (mmol/L) + 18.76 Difference between the kidney‐to‐L2 ratios (no unit)] (Table 2) served as a diagnostic test for determining whether UO was present; this had an AUROC of 0.908 (*P* < .001), with a sensitivity of 94.3%, a specificity of 78.6% and an optimal cutoff value of 42.90. We later generated a second formula with simplified coefficients adapted from the previous formula: [Log (odds of obstruction) = 1.2 Creatinine (mg/dL) + 7.0 Potassium (mmol/L) + 18.0 Difference between the kidney‐to‐L2 ratios (no unit)], as this formula also had an AUROC of 0.908 (*P* < .001), with the same sensitivity and specificity, and an optimal cutoff value at 41.3 (Figure 2).

**TABLE 2:** Univariate and multivariate odds ratios for predicting ureteral obstruction in BKLKS cats

Variables	Univariate analysis			Multivariate analysis		
	OR	95% CI	*P* value	OR	95% CI	*P* value
Serum creatinine (mg/dL)	1.296	1.08‐1.56	.006	1.188	0.96‐1.47	.11
Blood urea nitrogen (mg/dL)	1.019	1.00‐1.03	.01			
Phosphorus (mg/dL)	1.133	0.91‐1.41	.27			
Potassium (mmol/L)	5.729	1.70‐19.37	.005	7.286	1.38‐38.41	.02
Albumin (g/dL)	0.899	0.16‐5.15	.91			
Neutrophil‐to‐lymphocyte ratio	1.007	0.98‐1.03	.6			
Body temperature (°C)	1.418	0.36‐5.55	.62			
Body weight (kg)	0.791	0.49‐1.28	.34			
Length of big kidney (cm)	1.249	0.65‐2.39	.5			
Ratio of big kidney‐to‐L2	2.977	0.60‐14.89	.18			
Length difference (cm)	3.055	0.95‐9.80	.06			
Ratio difference	16.60	1.28‐216.08	.03	18.76	0.883‐398.73	.06

*Note*: The data were generated by binary logistic regression tests.

Abbreviations: BKLKS, big kidney‐little kidney syndrome; CI, confidence interval; L2, second lumbar vertebra; OR, odds ratio; Ratio difference, difference between the kidneys to second lumbar vertebra ratios.

Overall, 16 out of 36 obstructed BKLKS cats (44%) received surgical intervention (SI), and this consisted of 13 (36%) cats that received SUB surgery, 1 (2%) cat that received SUB surgery and hemodialysis treatment, 1 (2%) cat that received ureteral stent installation, and 1 (2%) cat that received ureteral stent installation and peritoneal dialysis treatment.

The degree of pelvic dilatation (before operation; *P* = .01) was significantly higher in the obstructed cats that had received SI than those that did not (median 1.17 [IQR 1.00‐1.41], n = 14 vs median 0.69 [IQR 0.51‐1.21], n = 20).

Among all the BKLKS cats, the difference in length between the kidneys was found to be linearly related to creatinine concentration (*R* = 0.316, *P* = .01) and BUN concentration (*R* = 0.280, *P* = .03), but was not linearly related to potassium concentration (*P* = .11), phosphorus concentration (*P* = .19), degree of pelvic dilatation (*P* = .6), or survival time (*P* = .74). Furthermore, in the UO cats, the degree of pelvic dilatation was not significantly linearly correlated with any of the renal‐related indices, including creatinine (*P* = .15), BUN (*P* = .14), phosphorus (*P* = .22), and potassium (with *P =* .98).

Using Spearman correlation, the length difference between the kidneys in all of the BKLKS cats was found to be significantly correlated with creatinine (*P* = .002) and BUN (*P* = .006), whereas in the UO cats, the degree of pelvic dilatation was not significantly correlated with any of the various renal‐related indices (Table 3).

**TABLE 3:** Correlation between kidney length differences and various variables among all BKLKS cats, and between pelvic dilatation degree and various variables among UO cats

Length difference^a^	Spearman correlation	*P* value	Pelvic dilatation degree^b^	Spearman correlation	*P* value
Pelvic dilatation degree (cm)^b^	−.149	.39	Length difference (cm)^a^	−.196	.27
Hematocrit (%)	−.180	.16	Hematocrit (%)	.055	.76
White blood cells (/μL)	−.112	.38	White blood cells (/μL)	−.199	.27
Blood urea nitrogen (mg/dL)	.342	.006	Blood urea nitrogen (mg/dL)	−.145	.41
Creatinine (mg/dL)	.387	.002	Creatinine (mg/dL)	−.198	.26
Potassium (mmol/L)	.187	.14	Potassium (mmol/L)	.080	.66
Phosphorus (mg/dL)	.273	.07	Phosphorus (mg/dL)	−.117	.53
Survival time (d)	.025	.85	Survival time (days)	.097	.59

Abbreviations: BKLKS, big kidney‐little kidney syndrome; UO, ureteral obstruction.

^a^ Measured using ventrodorsal abdominal radiography.

^b^ Measured using abdominal ultrasonography and the transverse plane of the obstructed kidney.

The median survival time of all BKLK cats was 254 days (range, 0‐1825 days).

Setting the survival cutoff at 30 days for the obstructed BKLKS cats, body temperature (BT) (rectal temperature; *P* = .009) taken at presentation was found to be significantly lower in those cats that did not survive (38.1 [37.9‐38.2]°C) than those that did survive (38.7 [38.3‐39.2]°C). The BUN (*P* = .04) and creatinine (*P* = .03) concentrations were also significantly higher in the nonsurvivor group than the survivor group (Table 4). When we compared the survival rate for 30 days after diagnosis, the UO cats that received SI (14/16 survived) did not show a significantly better 30‐day survival rate than that did not undergo surgery (15/20 survived) (*P* = .35). In addition, the former group (median 478 days [IQR 129‐1070 days]) did not have a significantly longer survival than the latter group (median 229 days [IQR 25‐482 days], *P* = .19).

**TABLE 4:** A comparison of selected variables between survivors and nonsurvivors for 30 days among all obstructed BKLKS cats

Variables	Survival (n = 30)			Nonsurvival (n = 7)			*P* value
	Median	IQR	n	Median	IQR	n	
Body temperature (°C)	38.7	38.3‐39.2	22	38.1	37.9‐38.2	5	.009
White blood cells (/μL)	10 050	7625‐13 775	28	11 600	9800‐24 400	7	.11
Neutrophils (/μL)	8209	5392‐12 049	28	12 943	8900‐22 461	6	.06
Lymphocytes (/μL)	1100	496‐1760	28	698	415‐1532	6	.56
Neutrophil‐to‐lymphocyte ratio	7.87	4.29‐15.13	28	24.15	8.35‐48.13	6	.19
Blood urea nitrogen (mg/dL)	91	36‐170	29	189	150‐252	7	.04
Creatinine (mg/dL)	9.00	3.1‐14.2	29	15.4	13.3‐17.4	7	.03
Surgical intervention	14/29 (48.3%)		29	2/7 (28.6%)		7	.35^a^
Survival time (d)	392	156‐1014	29	2	0‐6	7	<.001

Abbreviations: BKLKS, big kidney‐little kidney syndrome; IQR, interquartile range.

^a^ *P* value generated by chi‐square test, between survivor and nonsurvivor cats.

**FIGURE 3:**
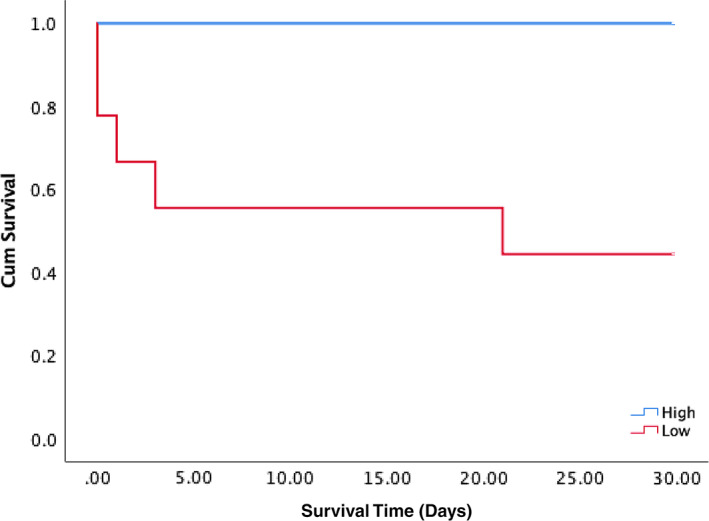
Receiver‐operating characteristic plots of diagnostic tests for ureteral obstruction using the formula [1.2 Creatinine (mg/dL) + 7.0 Potassium (mmol/L) + 18.0 Difference between the kidney‐to‐L2 ratios] (A). The formula which was designed as a diagnostic test for 30‐day survival in obstructed cats [1.0 BUN (mg/dL) + 0.8 Phosphorus (mg/dL) + 0.9 Body temperature (°C)] was presented with the ROC plot in (B). The area under the ROC (AUROC) for (A) is 0.908 (*P* < .001). Using a cutoff point of 41.3, for (A), the sensitivity was 94.3% and the specificity was 78.6%. The AUROC for (B) was 0.863 (*P* = .01). Using a cutoff point of 166.9, the sensitivity was 100% and the specificity was 78.9%. BUN, blood urea nitrogen; ROC, receiver operating characteristic

The univariate analysis results showed that BUN concentration (OR = 1.010) was significantly associated with whether a UO‐BKLK cat was able to survive to 30 days (Table 5), and an equation was generated for this using multivariate analysis: [Log (odds of death) = 1.029 BUN (mg/dL) + 0.783 Phosphorus (mg/dL) + 0.918 BT (°C)]. The formula served to establish a prognostic index as to whether a cat would survive to 30 days; this had an AUROC of 0.863 (*P* = .01), a sensitivity of 100%, a specificity of 78.9%, and an optimal cutoff value of 171.1. Other multivariate analysis models are shown in Table S2. We later generated a second formula with simplified coefficients adapted from the previous formula: [Log (odds of death) = 1.0 BUN (mg/dL) + 0.8 Phosphorus (mg/dL) + 0.9 BT (°C)], as this formula also had an AUROC of 0.863 (*P* < .01), with the same sensitivity and specificity, and an optimal cutoff value at 166.9 (Figure 2). When the obstructed BKLKS cats were divided into high‐value and low‐value groups based on either 1 of the formulas, the survival time was significantly different by log‐rank test between the 2 groups (*P* = .01) (Figure 3).

**TABLE 5:** Univariate and multivariate odds ratios for predicting death within 30 days for obstructed BKLKS cats

Variables	Univariate analysis			Multivariate analysis		
	OR	95% CI	*P* value	OR	95% CI	*P* value
Body temperature (°C)	0.177	0.02‐1.33	.09	0.918	0.86‐0.99	.02
BUN (mg/dL)	1.010	1.00‐1.021	.05	1.029	1.00‐1.06	.05
Creatinine (mg/dL)	1.072	0.97‐1.18	.17			
Phosphorus (mg/dL)	1.095	0.95‐1.26	.21	0.783	0.51‐1.21	.27
Potassium (mmol/L)	1.766	0.94‐3.31	.08			
Length difference (cm; Radiography)	1.098	0.42‐2.87	.85			
Size of renal pelvis (cm; Ultrasonography)	0.244	0.03‐2.56	.21			
Urine pH	0.114	0.01‐9.24	.33			

*Note*: The data were generated by binary logistic regression tests.

Abbreviations: BKLKS, big kidney‐little kidney syndrome; BUN, blood urea nitrogen; CI, confidence interval; OR, odds ratio.

## 4 DISCUSSION

In this study, we generated a cutoff value that will act as a reference when diagnosing cats with BKLKS. This involves calculating the length difference between 2 kidneys when observed on a ventrodorsal abdominal radiograph in clinically healthy cats. Radiography and ultrasonography have both been applied as adequate tools for assessing feline kidneys sizes. However, ultrasonography is also known for the disadvantage to subjectively obtain the longest kidney axis for each measurement as it relies heavily on user expertise, cat preparation, and the ultrasound machine models.^9^, ^10^ As a result, radiography was chosen as our main imaging modality for it provides more objective measurement of the kidneys and was not uncommon in our hospital. Furthermore, in practices where only ultrasonography is available, it might be impossible to obtain the length of the second lumbar vertebra for the assessment of kidney size and the absolute values of renal length differences can be also applied for the cats with ultrasonography examination. The data of the differences between the kidney‐to‐second‐lumbar‐vertebra‐ratios, measured on radiography, were also provided to give the information regarding relative sizes of the kidneys in cats with different sizes.

An equation was created for predicting the presence of UO before an ultrasonographic examination is performed, allowing practitioners to use other diagnostic modalities if ultrasonography is not available. Although several studies in the past have recorded the sizes of kidneys and the size differences between kidneys using abdominal radiographs,^11^, ^12^ as well as renal pelvic sizes before SI,^12^, ^13^ to our best knowledge, no study until the present 1 has demonstrated a correlation between such length measurements, disease prognosis, and the severity of azotemia. In this study, the length difference between kidneys was significantly larger in the UO group than that in the nonureteral obstruction (non‐UO) group. However, the length difference between kidneys was found to be correlated only with creatinine and BUN concentrations but not with the days of survival in BKLKS cats.

To date, no consistent criteria have been defined for diagnosing hydronephrosis using abdominal ultrasonography. This is partly because that in healthy cats, cats with chronic kidney disease (CKD), and cats with pyelonephritis, an increased renal pelvic diameter could also be found, while not all cases of outflow obstruction are associated with marked pelvic dilatation.^14^ In the past, 2 mm has been used as a cutoff value for the tentative diagnosis of UO.^11^ A report using pyelogram for definitive diagnosis of UO found the lower range for the affected pelvic diameter to be 3 mm by abdominal ultrasound.^15^ A more recent review article pointed out pelvic diameter > 3.5 mm in the transverse plane of the kidneys needs to be interpreted as potentially abnormal,^14^ and more severe cases of pelvic dilatation occurred in cats with urinary outflow obstruction.^14^ Although this value might still not be able to fully differentiate between UO and pyelonephritis, we decided to set the cutoff value for the presence of UO in this study at 3.5 mm, as it could have the potential to eliminate more cases in which CKD or mild pyelonephritis were the actual cause for the increased pelvic widths. However, in cases with the absence of evident UO on imaging findings and without the confirmation on antegrade pyelography or surgical exploration, false positive and false negative diagnosis on UO could still occur in our study design.

Similar to an earlier report stating that renal pelvic size is not associated with serum creatinine concentration,^16^ our results indicated that the increase in renal pelvic size among UO cats is not correlated with any renal‐associated variable, including creatinine, potassium, and phosphorus concentrations, although our study was underpowered in such calculations. Consequently, renal pelvis size and the length difference between a cat's kidneys need to be interpreted cautiously and should not be used alone to predict a prognosis and a clinicopathological abnormality; there is a clear need for additional information to help with any such diagnosis.

In this study, it was first observed that, among cats with BKLKS, when they are compared to control cats, there are significantly higher concentrations of creatinine and BUN, lower USG values, and a trend toward anemia being present. Furthermore, the UO cats, when compared with the non‐UO cats, have findings that are compatible with the characteristics of postrenal azotemia. Specifically, hypercreatininemia and hyperkalemia are common findings among cats with UO based on various previous studies.^12^, ^13^, ^15^

When the non‐UO group was compared with the control cats, there is also a statistical significant difference in creatinine concentrations. The lower USG in the non‐UO group also pointed toward the residual renal functioning of these cats being limited. Therefore, taking the clinicopathological abnormalities into account, non‐UO BKLKS cats might be likely to have CKD.

The leukogram of BKLKS cats showed significantly higher segmented cell counts, lower lymphocyte counts, and a higher NLRs when compared to control cats. Studies in human medicine have suggested that the NLR is possibly an early marker for detecting sepsis^17^ and community acquired pneumonia,^18^ as well as being an independent prognostic factor for patients with various types of cancers.^19^ Furthermore, this ratio also seems to be associated with renal and hepatic dysfunction in general.^20^ Although there has been no clear evidence of an association between NLR and veterinary medicine prognoses thus far, previous studies have pointed out that NLR could be useful when predicting a prognosis for canine soft tissue sarcoma,^21^ as well as when identifying systemic inflammation in dogs.^22^ In this study, the increased NLR in all of the BKLKS cats, along with increases in WBCs, possibly suggests the presence of inflammation.

Urine pH in the UO group was significantly lower than in the control cats. As stated, most UOs in cats are secondary to calcium oxalate uroliths.^4^ Furthermore, aciduria has been proposed as a risk factor for calcium oxalate formation.^23^ This result in our study supports the idea that there is a higher possibility of calcium oxalate ureterolithiasis in such cats, which is likely to contribute to UO, despite there being no direct evidence of this from stone analyses after surgery or at necropsy.

Subcutaneous ureteral bypass device is a viable treatment modality for cats with benign UO.^24^ However, in this study, it should be noted that, among the UO group cats with a pelvic size exceeding 0.5 cm, which is a prerequisite for a nephrostomy tube to be placed during SUB surgery,^25^ the 30‐day survival time difference between cats that have undergone SUB installation surgery, and those that have not had such an operation, was not significantly different. Specifically, there was no significant difference in overall survival between the 2 groups (data not shown). However, with the relatively small sample size and retrospective study design in the present study, as details specifying duration of previous azotemia, presence of other concomitant diseases and other variables could not be standardized, and that the surgical group had greater pyelectasia, implying a pre‐existing disease severity difference; the results must be interpreted with extreme caution. More in‐depth investigation and a stricter study design into the use of SUB devices in cats with UO should still be warranted.

Creatinine concentrations could correlate with true residual renal function and that this can help to predict the prognosis of cats with CKD.^26^, ^27^ However, during acute kidney injury or UO, the reduction in the renal function might be transient and is potentially reversible. Therefore, serum creatinine seems to be able to indicate only the degree of the current reduction in glomerular filtration rate in the kidneys, but cannot be used as a reliable prognostic factor.^28^ In this study, the results showed that, in UO cats, although there are significant differences in plasma creatinine concentrations between 30‐day survivors and nonsurvivors, creatinine values alone do not seem to be linked to the cats 30‐day survival using logistic regression modeling.

On the other hand, BUN concentrations do not seem to be a prognostic factor among cats with acute intrinsic renal failure,^29^ and in cats receiving hemodialysis,^30^ plasma BUN values are able to serve as an adequate prognostic factor for UO cats in our present study. The reason behind this difference is unclear; however, in recent human medical studies, a higher BUN has been associated with an increased risk of cardiovascular death in patients hospitalized for heart failure,^31^, ^32^ and in patients who suffer from death due to pneumonia.^33^ Whether the prognostic value of BUN in this study was due to similar properties is still unknown. Further investigations targeting this topic are necessary.

In the present study among UO cats, the BT of the nonsurvivor group was significantly lower than that of the survivor group. Although the mechanism of a lower BT in these cats with renal disease remains unclear, accumulating evidence based on human and veterinary studies has pointed out there is an increasing prevalence of hypothermia in uremic animals.^34^ This means that core BT might be able to serve as a prognostic index,^28^, ^35^ and this could lead to longer veterinary hospital stays for hypothermic cats with urethral obstruction.^36^

The present study has multiple limitations due to its retrospective nature. The renal sizes measured in the radiographs might have errors due to positioning differences between each cat. Furthermore, also in this context, interoperator variability might have affected the objectivity of the ultrasound measurements of the renal pelvis and ureters. It is quite hard to estimate the duration of an obstruction or the level of residual renal function before first presentation when acute azotemia or possibly UO is present. The fact that the control group had not been examined by abdominal ultrasonography could have resulted in inaccuracy when determining the various different characteristics between the control and the BKLKS cats. Furthermore, the sample sizes for the control group and for the SI group are quite small, and this could have affected the statistical significance of the group differences. A post hoc sample size determined that our study was underpowered (at a power of 80%), and the absence of correlation between the renal pelvic sizes and renal‐related indices could be a consequence of a type II error. In addition, symmetric dimethylarginine concentrations or direct glomerular filtration rates were not measured for these cats, and this could have limited our interpretations regarding renal function, and whether early or occult CKD existed in the control cats. The fact that not all of the BKLKS cats underwent urinalysis and abdominal ultrasonographic examination could have affected our understanding and interpretation of their renal function and the disease processes. There was no recording of whether any fluid therapy or diuretics were administered before the ultrasonographic examinations, which could possibly have transiently affected the cats' measurable renal pelvic size. Furthermore, in this study, no antegrade pyelography or computed tomography was performed to confirm the diagnosis of UO, and this might have resulted in false‐positive and false‐negative diagnoses for UO. The various etiologies of the UO, when present, could not be confirmed in every case due to the lack of surgery or necropsy results. Finally, it was also not possible to completely rule out cases with infiltrative renal diseases because surgical biopsy or necropsy was not performed on the cats. A strict prospective study designed to answer the questions would be strongly warranted in the future.

## CONFLICT OF INTEREST DECLARATION

Authors declare no conflict of interest.

## OFF‐LABEL ANTIMICROBIAL DECLARATION

Authors declare no off‐label use of antimicrobials.

## INSTITUTIONAL ANIMAL CARE AND USE COMMITTEE (IACUC) OR OTHER APPROVAL DECLARATION

Approved by National Taiwan University, NTU106‐EL‐00008.

## HUMAN ETHICS APPROVAL DECLARATION

Authors declare human ethics approval was not needed for this study.

## Supporting information


**Appendix** **S1**: Supporting information
