# Supplementary material for: Evaluation of and the prognostic factors for cats with big kidney‐little kidney syndrome
Source: J Vet Intern Med. 2021 Oct 15;35(6):2787–96. doi: 10.1111/jvim.16279 (PMC8692197; doi:10.1111/jvim.16279)
Supplement: Supplementary file 1 — Appendix S1: Supporting information [file JVIM-35-2787-s001.pdf]

Supplemental Table 1: Multivariate analysis for ureteral obstruction in all BKLKS cats using binary logistic regression

	Model1		Model 2		Model 3		Model 4		Model 5		Model 6	
Variables	OR	*P*	OR	*P*	OR	*P*	OR	*P*	OR	*P*	OR	*P*
Potassium	29.15	.023	11.74	.016	4.391	.021	5.737	.021	4.430	.022	7.286	.019
Blood urea nitrogen	1.000	.989			1.012	.115						
Creatinine	1.101	.754	1.173	.118			1.204	.093	1.240	.058	1.188	.111
Ratio of BK-to-L2	.013	.147										
Length difference	.000	.088	.000	.058			2.650	.147				
Ratio difference	2.202×10^{12}	.040	8.94×10^{8}	.039							18.76	.060
AUROC	.743	.008	.741	.009	.835	<.001	.906	<.001	.912	<.001	.908	<.001

AUROC, area under receiver operator curve; ratio difference, difference between the kidneys to second lumbar vertebra ratios; BK, big kidney; L2, second lumbar vertebra; OR, odds ratio

Supplemental Table 2: Multivariate analysis for 30-day survival in UO-BKLKS cats using binary logistic regression

	Model1		Model 2		Model 3		Model 4		Model 5		Model 6	
Variables	OR	*P*	OR	*P*	OR	*P*	OR	*P*	OR	*P*	OR	*P*
Body temperature	.851	.130	.918	.007	.931	.014	.862	.010	.915	.005	.918	.017
Blood urea nitrogen	1.041	.116	1.022	.091			1.008	.244	1.013	.039	1.029	.054
Creatinine	.714	.318	.886	.400	1.073	.489						
Phosphorus	.969	.918			1.055	.708					.783	.267
Potassium	2.054	.403					1.767	.178				
AUROC	.863	.014	.836	.021	.832	.025	.836	.021	.836	.021	.863	.014

AUROC, area under receiver operator curve; OR, odds ratio
